# Insights into the Mutation-Induced HHH Syndrome from Modeling Human Mitochondrial Ornithine Transporter-1

**DOI:** 10.1371/journal.pone.0031048

**Published:** 2012-01-26

**Authors:** Jing-Fang Wang, Kuo-Chen Chou

**Affiliations:** 1 Key Laboratory of Systems Biomedicine, Ministry of Education, Shanghai Jiao Tong University, Shanghai, China; 2 Shanghai Center for Bioinformation and Technology, Shanghai, China; 3 Gordon Life Science Institute, San Diego, California, United States of America; Consejo Superior de Investigaciones Cientificas, Spain

## Abstract

Human mitochondrial ornithine transporter-1 is reported in coupling with the hyperornithinemia-hyperammonemia-homocitrullinuria (HHH) syndrome, which is a rare autosomal recessive disorder. For in-depth understanding of the molecular mechanism of the disease, it is crucially important to acquire the 3D structure of human mitochondrial ornithine transporter-1. Since no such structure is available in the current protein structure database, we have developed it via computational approaches based on the recent NMR structure of human mitochondrial uncoupling protein (Berardi MJ, Chou JJ, et al. Nature 2011, 476:109–113). Subsequently, we docked the ligand L-ornithine into the computational structure to search for the favorable binding mode. It was observed that the binding interaction for the most favorable binding mode is featured by six remarkable hydrogen bonds between the receptor and ligand, and that the most favorable binding mode shared the same ligand-binding site with most of the homologous mitochondrial carriers from different organisms, implying that the ligand-binding sites are quite conservative in the mitochondrial carriers family although their sequences similarity is very low with 20% or so. Moreover, according to our structural analysis, the relationship between the disease-causing mutations of human mitochondrial ornithine transporter-1 and the HHH syndrome can be classified into the following three categories: (i) the mutation occurs in the pseudo-repeat regions so as to change the region of the protein closer to the mitochondrial matrix; (ii) the mutation is directly affecting the substrate binding pocket so as to reduce the substrate binding affinity; (iii) the mutation is located in the structural region closer to the intermembrane space that can significantly break the salt bridge networks of the protein. These findings may provide useful insights for in-depth understanding of the molecular mechanism of the HHH syndrome and developing effective drugs against the disease.

## Introduction

The hyperornithinemia-hyperammonemia-homocitrullinuria (HHH) syndrome (MIM 238970), also called “ornithine translocation deficiency”, is a rare autosomal recessive disorder, characterized by mental retardation, progressive spastic paraparesis, seizures, and myoclonus epilepsy [1], [2]. This kind of disease varies widely in its severity and age of onset. An infant with HHH syndrome may refuse to eat, or have poorly controlled breathing rate or body temperature. Some babies with this disorder may experience unusual body movements, or go into a coma 3,4. These disease signs and symptoms for most affected individuals do not appear until later in life. Late-onset forms of HHH syndrome are usually less severe than the infantile forms. Some people with late-onset HHH syndrome cannot tolerate high-protein foods. In some cases, high-protein meals or prolonged periods without food may cause ammonia to accumulate more quickly in the blood [5]. This rapid increase of ammonia may lead to episodes of vomiting, lack of energy, problems with coordination, confusion, or blurred vision. Complications of HHH syndrome may include developmental delay, learning disabilities, and stiffness caused by abnormal tensing of the muscles [3], [6].

The HHH syndrome is thought to be caused by the defective activities of the mitochondrial carrier responsible for transporting ornithine from the cytoplasm into the inner mitochondrial membrane [7]. Mutations of the solute carrier family 25 (mitochondrial ornithine transporter) member 15 (*SLC25A15*) gene (previously termed *ORNT1*) have been shown to be correlated with the HHH syndrome [8]. This gene encodes the mitochondrial ornithine transporter-1 that catalyzes the electroneutral exchange of ornithine for proton. The members of mitochondrial carrier family are mainly located in the inner mitochondrial membrane and allow exchange of substrates between the cytosol and the mitochondrial matrix. Mitochondrial carriers link the biochemical pathways of the cytosol and the mitochondrial matrix by transporting metabolites, nucleotides, inorganic ions, and vitamins across the mitochondrial inner membrane [9], [10]. These transport steps are essentially required for many biochemical processes, such as the synthesis of haem and iron-sulphur clusters, the synthesis of ATP from the oxidation of sugars and fats, production of heat, oxidative phosphorylation, amino acids and fatty acid synthesis and degradation, as well as the replication, transcription and translation of the mitochondrial genome [9], [10], [11].

Stimulated by the recent success in determining the 3D (three-dimensional) structure of the mitochondrial uncoupling protein 2 by NMR [12], the present study was initiated in an attempt to reveal the action mechanism of HHH syndrome by investigating the structures of wild-type and mutated mitochondrial ornithine transporter-1. In this study, we attempted to study the structures of wild-type and mutated mitochondrial ornithine transporter-1 by molecular modeling approaches, with an aim of providing detailed mechanism for HHH syndrome. As no structural information available in the protein structure databases, we firstly employed computational approach to construct a three-dimensional structural model for mitochondrial ornithine transporter-1. Subsequently, we employed flexible molecular docking operations to identify substrate binding site in mitochondrial ornithine transporter-1 followed by molecular dynamics simulations to optimize these structural models. Based on these computational studies, structural analysis was carried out to get detailed information for HHH syndrome.

## Materials and Methods

### 1. Molecular Modeling

**Figure 1:**
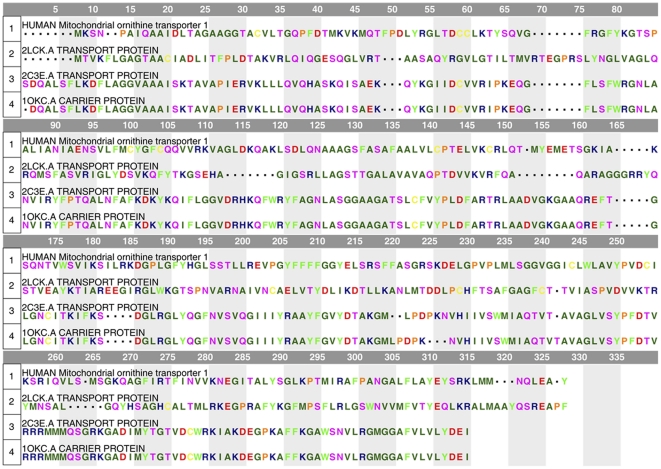
The multiple sequence alignment of the target protein with its three template structures. The target protein is human mitochondrial ornithine transporter-1; the three templates are 2lck.pdb, 2c3e.pdb, and 1okc.pdb. The amino acids are colored according to the following 7 types: (i) acidic, red; (ii) basic, dark blue; (iii) neutral hydrophilic, pink; (iv) aliphatic, green; (v) aromatic, light green; (vi) thiol-containing, yellow; (vii) imino, organ. The sequence similarity scores of the target protein with 2lck.pdb, 2c3e.pdb, and 1okc.pdb are 26%, 21%, and 20%, respectively.

The entire sequence of human mitochondrial ornithine transporter-1 (encoded by human *SLC25A15* gene), which contains 301 amino acids, was taken from the UniProt Database with an accession number of Q9Y619. The secondary structure of this protein was predicted by the membrane protein secondary structure prediction tool SPLIT4 [13]. To identify template proteins having similar folding structure or structural motif from Protein Data Bank, eight threading algorithms (MUSTER [14], FUGUE [15], HHSEARCH [16], PROSPECT [17], COMA [18], SP3 [19], SAM [20], and SPARKS [21]) were used to search the PDB library for those structures which have the similar sequence and secondary structures with the human mitochondrial ornithine transporter-1. The three top template hits obtained with the aforementioned eight threading algorithms were then selected to perform multiple sequence alignment (**Fig. 1**). Based on the template structures and multiple sequence alignment, the 3D structure of human mitochondrial ornithine transporter-1 was derived by the “segment matching” or “coordinate reconstruction” approach [22] and ab initio modeling [23]. The former was used to predict the aligned region structures based on the template fragments, whereas the latter was employed to construct the unaligned region structures. The segment matching approach had been successfully used to model the 3D structures of cytochrome P450 enzymes [24], [25], New Delhi metallo-beta-lactamse [26], and xylose reductases [27] before their crystal structures were available, timely providing useful information for both basic research and drug development in the relevant areas.

### 2. Molecular Docking

Computational docking operation is a useful vehicle for investigating the interaction of a protein receptor with its ligand and revealing their binding mechanism as demonstrated by a series of studies [28], [29], [30]. In this study, based on the computational structure model, molecular docking operations were carried out with Monte Carlo simulated annealing to get the favorable binding modes for human mitochondrial ornithine transporter-1 with L-ornithine. The present strategy consists of two stages: (1) development of the 3D structure of the protein receptor with the aforementioned techniques, and (2) investigation into the protein-ligand interactions by the techniques of molecular docking and molecular dynamics. A similar strategy has also been adopted by Gonzalez-Diaz et al. [31], [32], [33] recently. These authors used the program Hyperchem and/or the web server LOMETS [34] for stage (1). Furthermore, instead of molecular docking, they used the method MARCH-INSIDE to predict protein-ligand interactions (including protein-drug or protein-protein interactions) [31], [32], [33]. The method used by Gonzalez-Diaz et al. allowed them to develop new protein-ligand interaction servers like MIND-BEST, NL MIND-BEST, MISS-Prot, etc. However, these kinds of web servers could only yield the protein-ligand interaction scores for large-scale datasets without providing us with the detailed interactive geometry for some specific cases as concerned in the current study. The potential binding site was identified using Q-SiteFinder [35]. Since ligand binding in the active site might induce some conformational changes [36], [37], [38], a flexible docking procedure was adopted to construct the binding modes as described below. Before conducting molecular docking, we extracted 10,000 different configurations of human mitochondrial ornithine transporter-1 from a short-time molecular dynamics simulation as initial structures. The ligand was then docked into all these configurations to search for the favorable binding modes using the genetic algorithm, and the best 100 modes were taken for the further analyses. The docking program [39] used in this study would automatically generate a diversified set of configurations by randomly changing the atomic coordinates of the ligand. When a new configuration of the ligand was generated, the search for the favorable binding mode was operated within a specified 3D box by the simulated annealing to optimize the purely spatial contacts as well as electrostatic interactions. Finally, the favorable binding mode thus obtained was further optimized by a short-time molecular dynamics simulation. During the docking procedure, the Merck force field parameters were adopted, and the binding modes were assessed by a scoring function London dG [40].

### 3. Molecular Dynamics

Many marvelous biological functions in proteins and DNA and their profound dynamic mechanisms can be revealed by studying their internal motions [41], [42]. Likewise, to really understand the action mechanism of human mitochondrial ornithine transporter-1 with its ligands, we should consider not only the static structures concerned but also the dynamical information obtained by simulating their internal motions or dynamic process. In view of this, it is necessary to further perform a molecular dynamic simulation for the favorable binding mode obtained by the molecular docking operation as described above. Here the molecular dynamics simulations were performed by GROMACS 3.3.1 with GROMACS force field parameters [43], the periodic boundary conditions, and NPT ensembles. The topology file, force field parameters, as well as charges for the ligand were generated by online tool PRODRG [44]. Before starting simulations, the structural model was merged with a patch of a pre-equilibrated membrane with 256 DPPC bilayers from our previous studies [45], and subsequently solvated with explicit simple point charge (SCP) waters embedded in the simulation box. Meanwhile, sodium ions were randomly placed to neutralize the simulating system. The simulating system was then subjected to a 3000-step energy minimization with the steepest descents approach followed by conjugated gradient for the next 2000 steps. Finally, 1-ns molecular dynamics simulations were performed at 310 K. During our simulations, all bonds were constrained by the LINear Constraint Solver (LINCS) algorithm and atom velocities for start-up runs were obtained according to a Maxwell distribution at 310 K [46], [47]. To maintain the simulating system at a constant temperature and pressure, the Berdensen thermostat was applied with a coupling time of 0.1 and 1.0 ps [48]. The particle mesh Ewald algorithm was employed to treat electrostatic interactions with interpolation order of 4 and a grid spacing of 0.12 [49]. The van der Waals interactions were treated by using a cutoff of 12 Å. The integration step is 1 fs, and the coordinates were saved every 1 ps.

## Results and Discussion

### 1. Computational Model

**Figure 2:**
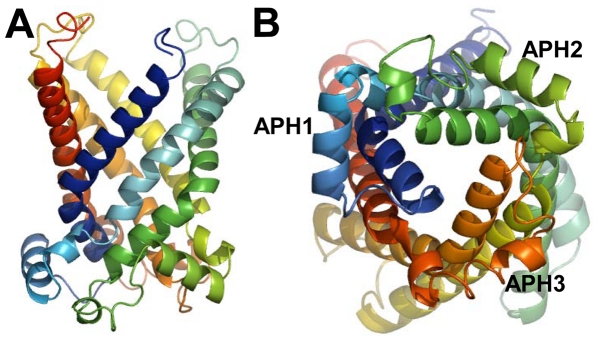
The 3D computational structure for the human mitochondrial ornithine transporter-1. (A) Viewed from the side to see more of its loops. (B) Viewed from the matrix side of the carrier to see more of its “repeat three”.

One of the most important procedures in modeling the 3D structure of a protein is how to identify or select the template structure. For the current case, none of the structure-known proteins in the Protein Data Bank has more than 30% sequence similarity with the human mitochondrial ornithine transporter-1. Under such a circumstance, it is not feasible to use the conventional homology modeling technique to derive the 3D structure of human mitochondrial ornithine transporter-1. To overcome such a difficulty, we resorted to the threading approach. After eight different threading operations were carried out, we found the following three template structures from the Protein Data Bank: (1) 2lck.pdb [12], (2) 2c3e.pdb [50], and (3) 1okc.pdb [51]. Subsequently, the sequences of the three template structures and the sequence of the human mitochondrial ornithine transporter-1 were subject to the multiple sequence alignment, as shown in Fig. 1. It was found from the alignment that the sequence similarity scores of the target protein with the three template proteins were 26% (for 2lck.pdb), 21% (for 2c3e.pdb), and 20% (for 1okc.pdb), respectively. In other words, none of the three structural templates has more than 30% sequence identity with the target protein. Under such a circumstance, we had to use the multiple templates, which could provide us with more guiding information for constructing the structure of human mitochondrial ornithine transporter-1. The concrete procedures were to divide the original sequence of human mitochondrial ornithine transporter-1 into a number of short segments with each containing 6 amino acids, followed by aligning these segments into all the three structural templates. Based on the sequence alignment of Fig. 1 and the template structures, the threading aligned region of the target protein was constructed. As for the threading unaligned region, the corresponding structure was derived with the ab initio approach. The entire 3D structure thus obtained for the human mitochondrial ornithine transporter-1 is shown in Fig. 2.

**Figure 3:**
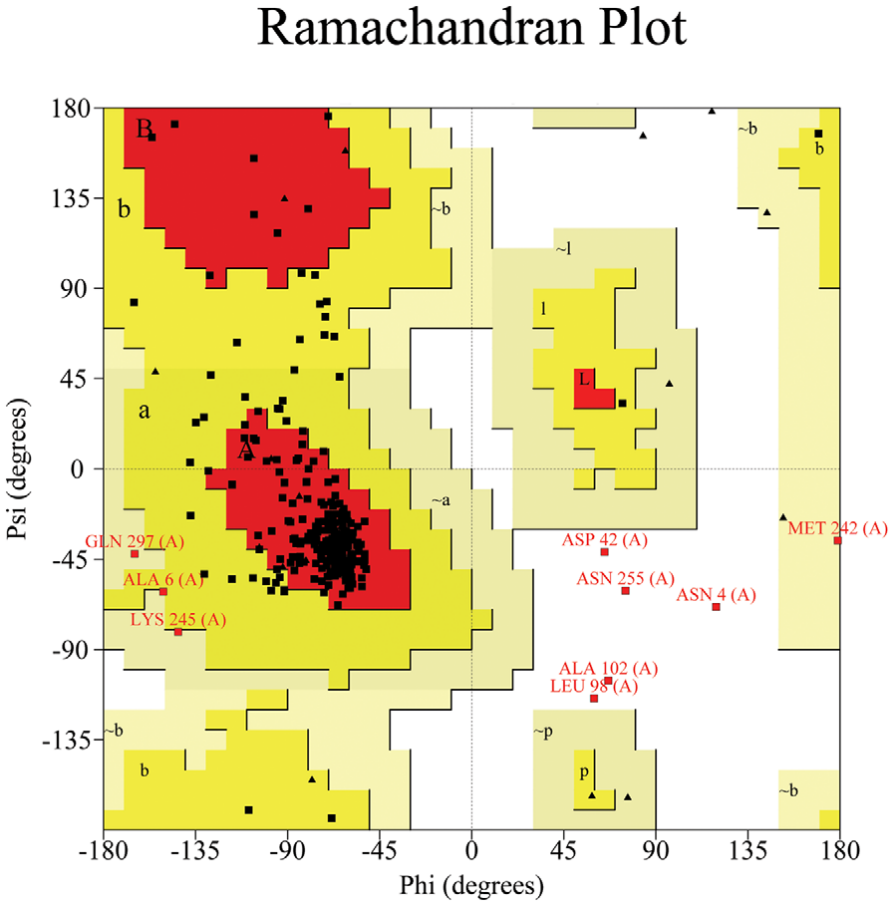
Ramachandran plot for the computational model of human mitochondrial ornithine transporter-1. It was derived from the plot that 90.4% residues located in the core region, 7.2% in the allowed region, 1.6% in the general region, and only 0.8% in the disallowed region. For a good quality model, the residues located in the core and allowed regions should be more than 90%, which is the case for our model since 90.4%+7.2% = 97.6%.

To validate the computational structure, the following examinations were performed. The stereochemistry and structural features were examined by PROCHECK [52]. It was indicated by PROCHECK that 90.4% residues of the computational structure were located in the “core” region, 7.2% in the “allowed” region, 1.6% in the “general” region, and only 0.8% in the “disallowed” region (Fig. 3). For a good quality model, the residues located in the core and allowed regions should be expected to be over 90%, which is the case for our model since 90.4%+7.2% = 97.6%. Moreover, for its main-chain residues, all the bond lengths and 94.0% of the bond angles are within the allowed limits. To further examine our computational model of human mitochondrial ornithine transporter-1, we also used the tool of QMEAN, which is a scoring function of a linear combination of 6 structural descriptors (Cβ interaction energy, all-atom pairwise energy, salvation energy, torsion angle energy, secondary structure, and solvent accessibility) [53]. The QMEAN score ranges between 0 and 1 with higher value to reflect a better quality of the input model. The QMEAN score for our current model is 0.682. Another tool ANOLEA [54] was also involved in examining the local model quality, and the result thus obtained was in good accord with the results by PROCHECK and QMEAN, indicating that most of the residues in the computational model were located in the favorable regions. Furthermore, we also used DFIRE [55] to evaluate the global model quality. Based on a distance-scaled finite ideal-gas reference state, DFIRE can be used to assess the non-bonded atomic interactions in the protein model. It can provide the pseudo energies for the computational model to reflect its quality. The result by DFIRE on our computational model was -366.03, indicating that it was quite close to the native conformation. All these validated results indicate that our computational model is quite reliable.

### 2. Substrate Binding

As expected, the computational structure is a channel-like one (Fig. 2A), in which the three pseudo-repeats adopted the similar folding pattern as those in the human UCP2 [12] and bovine ADP/ATP carrier [51]. Each pseudo repeat contained a transmembrane helix (TMH), a loop, an amphipathic helix (APH), and a second transmembrane helix (Fig. 2B). The structural similarity with the human UCP2 and bovine ADP/ATP carrier further indicates that the members in the mitochondrial carrier family have a conserved folding structure and that the variations within this conserved fold frame would govern the specificity of substrate binding and translocation.

**Figure 4:**
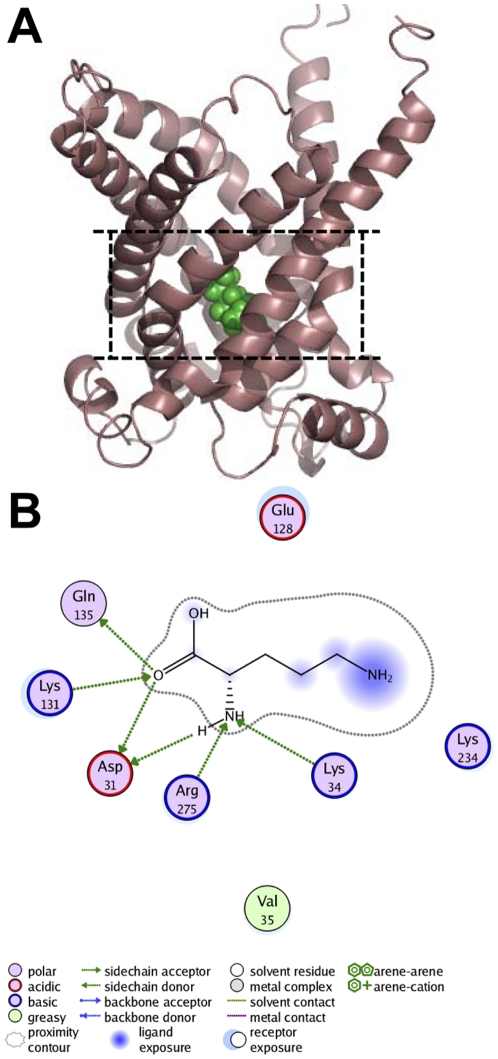
Binding model of L-ornithine bound to human mitochondrial ornithine transporter-1. (A) Lateral view of the ornithine binding interactions, where the backbone of human mitochondrial ornithine transporter-1 is colored in coffee, while the ligand L-ornithine in green. (B) A close view of the binding interactions for more detailed information.

Based on the computational model, we docked L-orinithine into human mitochondrial ornithine transporter-1 with the scoring function of London dG to assess the binding interactions, and the most favorable binding mode thus found is shown in Fig. 4A. Among the 10,000 configurations investigated for the protein, only the binding mode was selected that had the correct chemistry and geometry for it to bind the substrate. The corresponding binding pocket thus identified is quite similar to the ones in yeast mitochondrial carriers that were reported to have the conserved substrate binding sites [56], [57]. Like the yeast mitochondrial carriers, the residues involved in such substrate-binding mode were quite conservative in these homologous carriers since they, although from different organisms, have the same function [57]. It is interesting to see from the most favorable binding mode of human mitochondrial ornithine transporter-1 with L-orinithine that six remarkable hydrogen bonds were observed (Fig. 4B). For the detailed information of the six hydrogen bonds, see Table 1. These hydrogen bonds, especially the one formed by Arg275, were thought to play crucial roles for the substrate in recognizing its receptor and performing proper binding.

**Table 1:** Detailed information for the hydrogen bonding formed by human mitochondrial ornithine transporter-1 and its substrate L-ornithine.

H-donor	H-receptor	Bond dist (Å)	Lifetime (%)
L-ornithine (O_β_)	Asp31	2.17	31.1
L-ornithine (O_β_)	Gln135	2.67	33.9
Arg275	L-ornithine (NH_2_)	2.56	40.4
Lys131	L-ornithine (O_β_)	2.86	56.0
L-ornithine (NH_2_)	Asp31	2.55	73.0
Lys34	L-ornithine (NH_2_)	2.60	96.8

### 3. Mutations in HHH Syndrome

**Figure 5:**
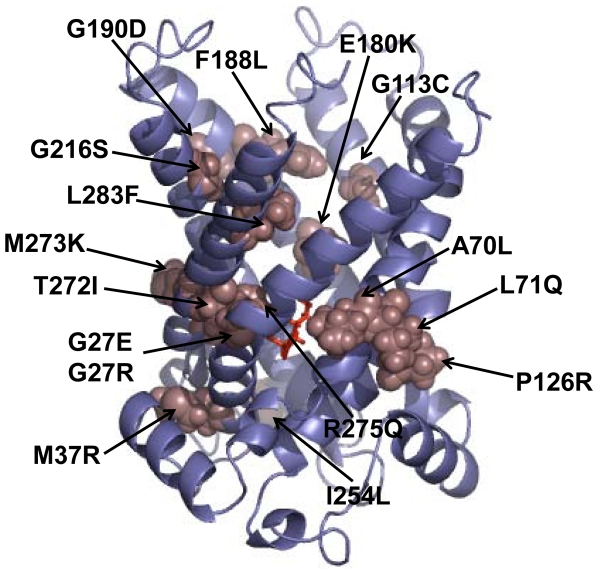
A schematic drawing to summarize the 16 missense mutations of human mitochondrial ornithine transporter-1 in the HHH syndrome. The backbone structure of the protein is colored in purple with the ribbon drawing, while its mutation parts in coffee with the spacing filling drawing. The L-ornithine is in red with the stick drawing.

A total of 16 missense mutations involving 15 amino acids within human mitochondrial ornithine transporter-1 have been reported from the HHH syndrome patients [8], [58], [59], [60], [61], [62], [63], [64]. Shown in Fig. 5 is the distribution of the aforementioned 16 missense variants when mapped onto our computational model. Based on the aforementioned computational analysis, these 16 missense variants can be divided into three classes. The first class of the mutations occurs in the three pseudo-repeats (M37R and I254L). The disease-causing mutations in this category may change the folding structure of the pseudo-repeats that are conserved in the mitochondrial family, further altering the structure of mitochondrial matrix to cause the dysfunction of the protein. The mutations in the second class (G27E/G27R, A70L, L71Q, P126R, E180K, T272I, M273K, and R275Q) may directly affect the structures of substrate binding pocket. G27E/G27R, A70L, and L71Q may have a deleterious impact on the hydrogen bonding of Asp31, Lys34, Lys131, and Gln135. R275Q can break a significant hydrogen bond, which may also be influenced by T272I and M273K. The third class of mutations is involved with G113C, F188L, G190D, G216S, and L283F. These residues are located in the cytoplasmic domain near the lipid bilayers, and are thought to form salt bridge networks to control the channel opening and substrate translocation [65].

In conclusion, we have developed a 3D structure for human mitochondrial ornithine transporter-1 based on 2lck.pdb, the NMR structure of human mitochondrial uncoupling protein 2, as well as 2c3e.pdb and 1okc.pdb, the crystal structures of the bovine ADP/ATP carriers. The examinations by PROCHECK and QMEAN have indicated that the computed structure is quite reliable. It was observed by docking L-ornithine into the human mitochondrial ornithine transporter-1 that the binding mode was featured by six remarkable hydrogen bonds between the receptor and ligand. The relationship between the mutations of human mitochondrial ornithine transporter-1 and the HHH syndrome was analyzed. According to their different locations, the disease-causing mutations can be categorized into the following three types: the first type is that the mutation occurs in the pseudo-repeat regions so as to change the mitochondrial matrix structure of the protein; the second type is directly affecting the substrate binding pocket so as to reduce the substrate binding affinity; the third type is for the mutation located in the cytoplasmic domain that can significantly break the salt bridge networks of the protein. These findings may provide useful insights for understanding the molecular mechanism of causing the HHH syndrome.
